# Mechanism of Signalling and Adaptation through the *Rhodobacter sphaeroides* Cytoplasmic Chemoreceptor Cluster

**DOI:** 10.3390/ijms20205095

**Published:** 2019-10-14

**Authors:** Jennifer A. de Beyer, Andrea Szöllössi, Elaine Byles, Roman Fischer, Judith P. Armitage

**Affiliations:** 1 Department of Biochemistry, University of Oxford, Oxford OX1 3QU, UK; jennifer.de-beyer@csm.ox.ac.uk (J.A.d.B.); andrea.szollossi@gmail.com (A.S.); elaine.byles@bioch.ox.ac.uk (E.B.); 2 Nuffield Department of Medicine, University of Oxford, Oxford OX3 9DU, UK; roman.fischer@ndm.ox.ac.uk

**Keywords:** chemotaxis, *Rhodobacter*, adaptation, chemoreceptors, cytoplasmic signalling, mass spectrometry, signal transduction, signal integration, methyl-accepting chemotaxis proteins

## Abstract

*Rhodobacter sphaeroides* has two chemotaxis clusters, an *Escherichia coli*-like cluster with membrane-spanning chemoreceptors and a less-understood cytoplasmic cluster. The cytoplasmic CheA is split into CheA_4_, a kinase, and CheA_3_, a His-domain phosphorylated by CheA_4_ and a phosphatase domain, which together phosphorylate and dephosphorylate motor-stopping CheY_6_. In bacterial two-hybrid analysis, one major cytoplasmic chemoreceptor, TlpT, interacted with CheA_4_, while the other, TlpC, interacted with CheA_3_. Both clusters have associated adaptation proteins. Deleting their methyltransferases and methylesterases singly and together removed chemotaxis, but with opposite effects. The cytoplasmic cluster signal overrode the membrane cluster signal. Methylation and demethylation of specific chemoreceptor glutamates controls adaptation. Tandem mass spectroscopy and bioinformatics identified four putative sites on TlpT, three glutamates and a glutamine. Mutating each glutamate to alanine resulted in smooth swimming and loss of chemotaxis, unlike similar mutations in *E. coli* chemoreceptors. Cells with two mutated glutamates were more stoppy than wild-type and responded and adapted to attractant addition, not removal. Mutating all four sites amplified the effect. Cells were non-motile, began smooth swimming on attractant addition, and rapidly adapted back to non-motile before attractant removal. We propose that TlpT responds and adapts to the cell’s metabolic state, generating the steady-state concentration of motor-stopping CheY_6_~P. Membrane-cluster signalling produces a pulse of CheY_3_/CheY_4_~P that displaces CheY_6_~P and allows flagellar rotation and smooth swimming before both clusters adapt.

## 1. Introduction

Chemotaxis, the ability of motile bacteria to move along chemical gradients to reach nutrients, is well understood in *Escherichia coli*. However, the *E. coli* chemotaxis system is relatively simple when compared with those of other bacterial species, such as *Rhodobacter sphaeroides* [1,2].

In *E. coli*, transmembrane chemoreceptors (methyl-accepting chemotaxis proteins; MCPs) arranged in large hexagonal arrays of trimers of dimers signal changes in nutrient or toxin concentrations to crosslinking CheA histidine kinases [3]. A reduction in nutrient concentrations causes CheA to phosphorylate a small diffusible protein, CheY. CheY~P binding to the flagellar motor causes the motor to change rotational direction, triggering a transient tumble. Adaptation to any environmental change is critical if bacteria are to respond to future changes. The receptors adapt to the new background concentration because the activity of CheB, a methylesterase, is also controlled by CheA. CheB~P removes methyl groups from key glutamates on the chemoreceptor, resetting its signalling state. A methyltransferase, CheR, then slowly resensitises the chemoreceptor by remethylating the sites, allowing the chemoreceptor to respond to future change [4]. 

**Figure 1:**
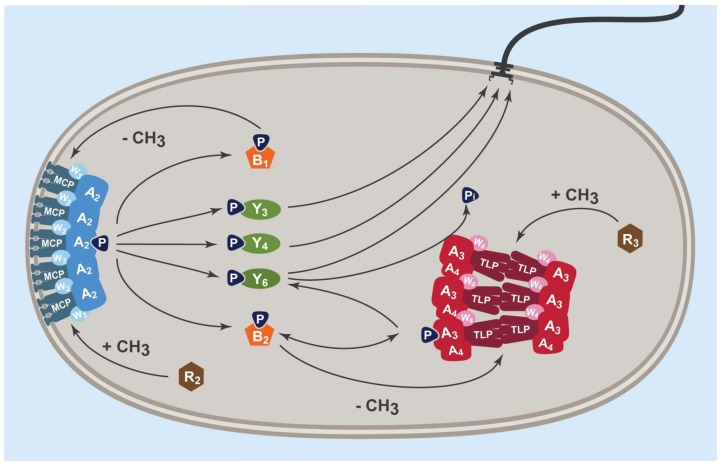
*R. sphaeroides* chemosensory proteins and CheA and CheW homologues localise to two areas in the cell, forming membrane-associated clusters (MCPs, CheA_2_, CheW_2_, and CheW_3_) and cytoplasmic clusters (Tlps, CheA_3_, CheA_4_, and CheW_4_). CheR_2_ also localises to the membrane cluster and CheR_3_ to the cytoplasmic cluster. CheB and CheY homologues are diffuse within the cell. Phosphotransfer shown is known from in vitro studies [12]. Methyltransfer shown is hypothesised based on CheR localisation and *cheB* gene positions (CheB_1_ is encoded on the membrane cluster operon and CheB_2_ on the cytoplasmic cluster operon).

*R. sphaeroides* swims using a single stop-start motor [5]. It controls the frequency of stopping using signals from two chemotaxis pathways, each encoded on a different operon (Figure 1). One array lies in the membrane and is very similar to that characterised in *E. coli*. The second lies in the cytoplasm and is less well-understood [6,7,8,9]. Previous work has suggested there are two chemoreceptor-like proteins in the cytoplasmic cluster, TlpC and TlpT. TlpT is closest to a canonical chemoreceptor; it has the highly conserved signalling domain of an MCP but lacks the transmembrane domain. Its associated histidine kinase is a CheA-like protein split into two, one with the ATP-binding kinase P4 domain, CheA_4_, and the other with a conserved P1 histidine domain, CheA_3_ [10,11]. The two CheAs have very different receptor-binding domains (P5), suggesting that they each form a different complex with the receptors. CheA_3_ also contains an unusual domain that can dephosphorylate CheY_6_~P, but no other CheY~P. In contrast, TlpC does not contain a typical MCP transmembrane domain, sensory domain, or the glycine turn motif usually used to mark the centre of the highly conserved signalling domain. 

*R. sphaeroides* chemotaxis occurs through changes in stopping frequency. The membrane cluster operon encodes two CheYs (CheY_3_ and CheY_4_) and the cytoplasmic cluster operon one (CheY_6_). CheY_6_ and either CheY_3_ or CheY_4_ are required for chemotaxis, although only CheY_6_ stops the motor [13]. Deleting both CheY_3_ and CheY_4_ results in wild-type swimming but no chemotaxis. CheY_6_~P is required to stop the motor and is phosphorylated and dephosphorylated by CheA_3_. The phosphorylation state of the CheA_3_ histidine depends on CheA_4_ activity. Two adaptation proteins, CheB_2_ and CheR_3_, are also associated with the cytoplasmic cluster [14]. 

Cryo-electrontomography showed that the cytoplasmic chemoreceptors are arranged in hexagonal arrays with the same organisation as the membrane-spanning chemoreceptors, but with the heads of the receptors overlapping to form a sandwich. The conservation of the receptor arrangement between the membrane-spanning and cytoplasmic chemosensory pathways suggests that this arrangement is critical for signalling changes in the extra- and intra-cellular environment [3,15]. 

The cytoplasmic cluster is essential for chemotaxis, as deletion of any of the key proteins involved in its assembly causes loss of chemotaxis [16]. Deletion of CheR_3_, which localises to the cluster, also causes loss of chemotaxis and smooth swimming, suggesting that adaptation is also critical [14]. Very little is known about the mechanism by which the cytoplasmic chemoreceptors signal and adapt, or whether they are controlled by one or both of the kinase domains. TlpC is encoded with the proteins forming the membrane-associated cluster, but localises to the cytoplasmic cluster [6]. Deletion causes the cluster to become more diffuse. TlpT is encoded in the operon encoding the chemosensory proteins of the cytoplasmic cluster. No cluster forms if TlpT is deleted, but the cluster will form if TlpT is expressed from a plasmid, showing it has a critical role in organising the chemosensory cluster [17]. It also interacts with the key protein PpfA, a ParA homologue involved in positioning the cluster on the chromosome surface, ensuring each cell inherits a cluster on division [18,19]. 

TlpT is the most likely candidate for methylation, as it is vital for chemotaxis and is classified as a 36H receptor based on the length of its highly conserved domain. Methylation has only been observed in 44H receptors (such as McpH in *Bacillus subtilis*) and 36H receptors (such as Tsr in *E. coli*) [20]. However, bioinformatics has not identified canonical methylation sites in TlpT’s cytoplasmic domain.

Using nano liquid chromatography coupled tandem mass spectrometry (nLC-MS/MS) and mutagenesis, we identified deamidation and methylation sites on TlpT and found that these sites are involved in adaptation. By combining protein sequence coverage enhanced mass spectrometry with phenotype studies and analysis of protein-protein interactions using bacterial two-hybrid studies [21], we have developed a model for the control of the single flagellar motor by two chemosensory pathways and show that the cytoplasmic cluster controls the strength of any chemosensory response.

## 2. Results

### 2.1. Structure of the Cytoplasmic Cluster

Table 1 shows the results of a bacterial two-hybrid analysis to investigate the interactions between the cytoplasmic cluster proteins. TlpT and TlpC interacted with themselves but not each other. Both interacted with CheW_4_, a key component in chemosensory array assembly. Although TlpT interacted with CheA_3_ and CheA_4_, TlpC only interacted with CheA_3_. The input signal to the two domains of the histidine kinase may thus be different, with TlpT responding to chemoeffectors and regulating CheA kinase activity and thus the strength of any response through CheY_6_~P production. Signals through CheA_3_ are likely to only regulate the dephosphorylation rate of CheY_6_~P.

### 2.2. Steady State and Dynamic Phenotype of CheB and CheR Deletions

**Figure 2:**
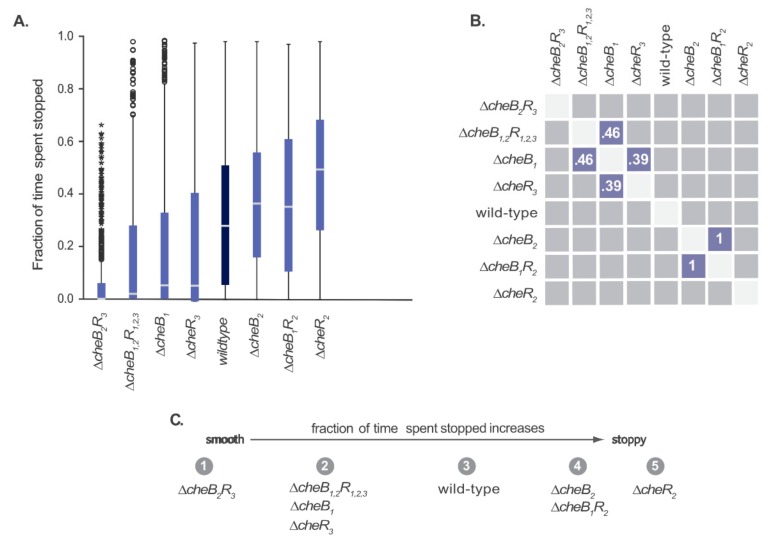
Steady-state swimming phenotypes of methylesterase and methyltransferase deletions in *R. sphaeroides*, calculated from tracks collected over 18 min of free swimming video (9 × 2 min), after censoring (see Methods 4.8). Table 2 shows the number of tracks analysed. (**A**) Box plots showing the distribution of fraction of time spent stopped. The box contains the interquartile range (middle 50% of values; IQR), the centre line indicates the median, and the whiskers indicate the minimum and maximum values no smaller/greater than 1.5 × IQR. Circles are outliers 1.5–3.0 × IQR and stars are extreme values more than 3.0 × IQR. (**B**) Post-hoc pairwise comparisons between distributions of fraction of time spent stopped for each mutant (following a Kruskal Wallis test with *p* < 0.001). Numbers indicate the *p*-value for that comparison. Comparisons not shown are *p* < 0.001. Blue squares indicate pairs that are not significantly different from one another (*p* ≥ 0.05). (**C**) Strains grouped based on the proportion of time spent stopped, as judged by pairwise comparisons, from smooth swimming to very stoppy.

The steady-state swimming behaviour of CheB and CheR mutants was characterised by recording their tracks and calculating the amount of time the cells spent stopped. Krusal-Wallis test with post-hoc Dunn pair-wise comparison test showed that all of the mutants differed from wild-type (Figure 2). 

The mutants’ chemotaxis response to addition and removal of attractant was tested by tethering. The mutant strains were tethered by their flagella to a glass slide without attractant for 3 min. Propionate was then flowed through for 5 min, then removed and cells observed for another 5 min. Supplementary Figure S1 shows the output for each possible phenotype; wild-type: stops rotating on removal of attractant then adapts, returning to prestimulus behaviour; unresponsive: continues to rotate on addition and removal of attractant; inverted: begins rotation on addition of attractant; inverted adaptive: begins rotation on addition of attractant and stops after a period; and responsive: stops rotating on removal of attractant and does not rotate again. Table 2 summarises the results of both experiments. 

Deletion of the methyltransferases and methylesterases encoded in the membrane and cytoplasmic clusters caused opposite phenotypes. Deleting CheB_1_ caused a smooth swimming non-chemotactic phenotype. Deleting CheB_2_ caused a stoppy phenotype with reverse adaptive behaviour: most cells started to rotate smoothly on addition of attractant and stopped before or after attractant was removed, not regaining movement again. Deleting CheR_2_ caused a stoppy non-chemotactic phenotype, while CheR_3_ deletion produced a smooth non-chemotactic phenotype. 

Deleting CheR_2_ and CheB_2_ caused stoppy cells while CheR_3_ and CheB_1_ deletion caused smooth swimming, suggesting the effect of adaptation is to produce opposite responses in the two sets of receptors. In both cases the effect of deleting the transferase was dominant. Smooth swimming resulted from deleting all CheR homologues, while a stoppy phenotype resulted from deleting all CheBs. Deleting all adaptation proteins also resulted in smooth swimming, suggesting that signalling through the chemosensory cluster is dominant. In comparison, deleting CheR in *E. coli* causes smooth swimming, deleting CheB causes tumbly behaviour, and deleting both results in a return to a wild-type bias at steady state [22].

Having established that adaptation was critical through both pathways, we tried to identify the adaptation sites on the cytoplasmic chemoreceptors. We did not identify any possible glutamates with the bioinformatics approach of Alexander and Zhulin [20]. Mutagenesis of glutamates in their suggested regions produced no behavioural phenotype. We therefore developed a method for identifying adaptation sites using mass spectrometry, as mass spectrometry had been successfully used before [23].

### 2.3. E. coli Tsr MS-MS Results

We first tested whether the devised method could identify the four known adaptation sites in Tsr isolated from *E. coli* with limited false positives and negatives. 

We purified C-terminal His-tagged Tsr expressed in wild-type *E. coli* and in a strain gutted for all adaptation genes in duplicate. The chemoreceptors were digested with elastase to maximise protein sequence coverage [24] and subjected to MS/MS. We obtained 89% coverage of Tsr in the wild-type strain and 84% coverage in the gutted strain. We then quantified methylation of glutamates or deamidation and methylation of glutamines in the peptides. 

Seven glutamates and fourteen glutamines were found to be modified, including all four known adaptation sites, indicating that the modifications of interest survived sample preparation (Supplementary Table S1). The lowest abundance of modified peptide in a known adaption site was 10% for E304, then E493 (12.5%), Q297 (24.31%), and Q311 (64.29%). Site E493 was poorly resolved from its glutamate pair partner, E492 (37.5% modified), which is a common problem in MS/MS. We therefore considered the pair together.

We compared the extent of modification in peptides from the wild-type strain, where chemotactic adaptation could occur, with peptides from the gutted strain, where adaptation could not occur. As peptide intensity measurements do not necessarily correlate with abundance, we counted the relative occurrence of modified sequences for a modification site, compared with unmodified sequences. The total protein abundance in each sample was used to normalise each peptide abundance, to allow direct comparison of relative abundance across the four samples.

**Figure 3:**
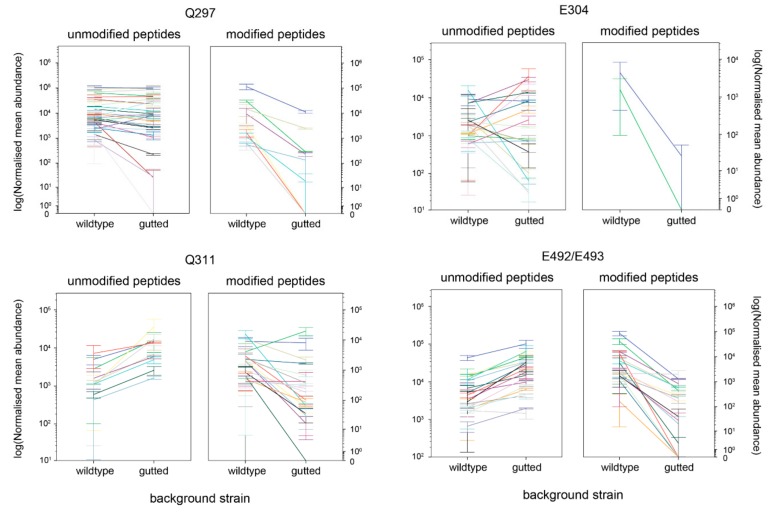
Relative abundance of each *E. coli* Tsr peptide (modified or unmodified) in the two backgrounds, RP437 (wild-type) and RP1091 (gutted of chemotaxis genes) identified in MS/MS that contains a glutamate or glutamine site known to be adaptation sites. Sites E492 and E493 are combined, as it is difficult to resolve data for a glutamate pair. Each measurement was in duplicate, with error bars showing the two values. Each colour within a plot represents one peptide containing that residue.

Supplementary Figure S2 shows the relative abundance of each peptide (modified or unmodified) in the two backgrounds, for those sites that were modified in 10% or more of the identified peptides, while Figure 3 shows just those known to be adaptation sites. There was no clear difference between relative abundance of peptides known to not be involved in adaptation in the two backgrounds of modified and unmodified peptides at the log scale (Q318, E321, and E325). In comparison, peptides containing the four known adaptation sites in Tsr showed clear regulatory patterns, with much greater abundance of methylated and deamidated peptides in the wild-type background than in the gutted background and much greater abundance of unmodified peptides in the gutted background. Thus, using regulatory patterns across different backgrounds offers a clear way to screen out falsely identified possible adaptation sites. 

### 2.4. Identification of Adaptation Sites of TlpT

C-terminal His-tagged TlpT (TlpT-His) was expressed from an expression plasmid (pIND_4_-TlpT-His) in three *R. sphaeroides* strains, wild-type, a background strain with no CheB homologues, and a background strain with no CheR homologues. *CheBRA*, a putative methyltransferase-methylesterase fusion gene, was also deleted from both background strains. The plasmids were induced using very low levels of IPTG to produce low levels of tagged chemoreceptor that would integrate into the chemotaxis clusters and avoid the formation of inclusion bodies. As the Tsr yield had been low, a concentration step was added for TlpT. Three backgrounds were used to improve the ability to identify regulatory patterns. The protein purification and MS/MS protocol otherwise proceeded as for Tsr, in triplicate. 

97% coverage of TlpT was obtained in all three *R. sphaeroides* strains, due to the added concentration step. The total protein abundance in each sample was used to normalise each peptide abundance, to allow direct comparison across the nine samples.

**Figure 4:**
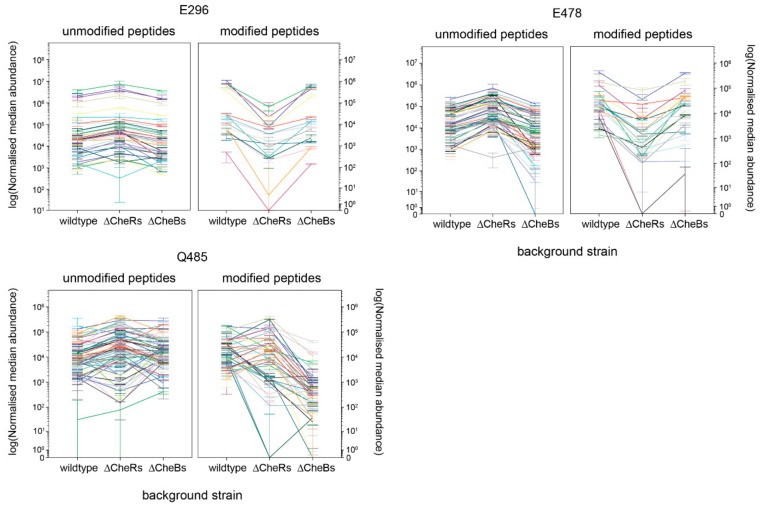
Relative abundance of each *R. sphaeroides* TlpT peptide (modified or unmodified) in the three backgrounds, WS8N (wild-type), JPA3265 (all CheB homologues deleted) and JPA2366 (all CheR homologues deleted), for glutamate and glutamine sites with modifications in over 5% of identified peptides that showed a regulatory pattern consistent with adaptation. Each measurement was done in triplicate, with the median shown and error bars showing min and max. Each colour within a panel represents one peptide containing that residue.

Table S2 shows the number of peptides containing glutamate and glutamine with and without modification. Ten glutamates and eight glutamines were modified. Those that were modified in more than 5% of peptides were examined for regulatory patterns. A lower threshold was used than had captured all known sites in Tsr (10% modification) to ensure we did not miss a potential site. Only regulatory patterns visible at the log scale were considered significant. Figure 4 shows those sites with a clear regulatory pattern consistent with *R. sphaeroides* adaptation (Supplementary Figure S3 shows results for all residues with over 5% modified peptides). 

Glutamate sites E296 and E478 showed a pattern consistent with adaptation, with a decrease in abundance of modified (methylated) peptide in the background with no CheRs, compared with the wild-type and no CheB backgrounds. Glutamine site Q485 showed a pattern consistent with adaptation, with a decrease in abundance of modified (all only deamidated) peptide in the no CheB background compared with the wild-type and no CheR backgrounds. 

### 2.5. Comparison with Known Adaptation Sites

Known adaptation sites follow a consensus sequence identified by Alexander and Zhulin [20], [ASTG]-[ASTG]-X-X-[EQ]-[EQ]-X-X-[ASTG]-[ASTG], with methylation typically on the second residue in the EQ-EQ pair, which is heptad position **c**. All three candidate adaptation sites on TlpT identified with MS-MS (E296, E478, and Q485) lie at position **c** in a heptad, which agrees with the register for the consensus sequence. However, only one of these sites, E296, is the second residue in a canonical EQ/EQ pair, identified in most studied chemoreceptors. E478 is in a DE pair and Q485 in a TQ pair. Using a relaxed version of the Alexander and Zhulin consensus sequence that allowed DE and TQ pairs identified another potential site, E289. 

### 2.6. Testing the Candidate Adaptation Sites

The four candidate adaptation sites were mutated to alanine individually, in pairs, and quadrupally. All single mutants, one pair (TlpT E289A E296A), and the quadruple mutant were successful. Strains are listed in Materials and Methods.

The mutants were tested for normal formation of the cytoplasmic chemosensory cluster by expressing CheW_4_ tagged with N-terminal YFP in each strain, as TlpT was shown to interact with CheW_4_ in the bacterial two-hybrid experiments, and CheW_4_ localises to the cytoplasmic cluster and is delocalised in TlpT deletions. Fluorescent foci indicating clusters were observed in over 90% of cells in all tagged strains (Supplementary Table S3 and Figure S4), including the double mutant JPA2368 (TlpT E289A, E296A) and the quadruple mutant JPA2371 (TlpT E289A, E296A, E478A, Q485A). As none of the putative site mutations affected cluster formation, any phenotypes observed in the site mutants are probably the result of changes in adaption, not cluster formation.

The effects of the mutations on steady state swimming behaviour and responses to addition and removal of a chemoattractant were then tested. 

### 2.7. TlpT Mutant Swimming under Steady-State and Dynamic Conditions

**Figure 5:**
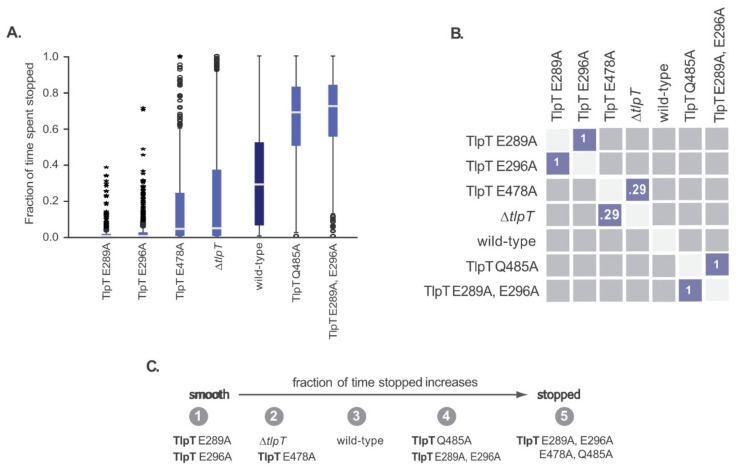
Steady-state swimming phenotypes of TlpT mutants in *R. sphaeroides*, calculated from tracks collected over 18 min of free swimming video (9 × 2 min), after censoring (see Methods 4.8). Table 3 shows the number of tracks analysed. (**A**) Box plots showing the distribution of fraction of time spent stopped. The box contains the interquartile range (middle 50% of values; IQR), the centre line indicates the median, and the whiskers indicate the minimum and maximum values no smaller/greater than 1.5 × IQR. Circles are outliers 1.5–3.0 × IQR and stars are extreme values more than 3.0 × IQR. (**B**) Post-hoc pairwise comparisons between distributions of fraction of time spent stopped for each mutant (following a Kruskal Wallis test with *p* < 0.001). Numbers indicate the *p*-value for that comparison. Comparisons not shown are *p* < 0.001. Blue squares indicate pairs that are not significantly different from one another (*p* ≥ 0.05). (**C**) Strains grouped based on the proportion of time spent stopped, as judged by pairwise comparisons, from smooth swimming to very stopped.

The steady-state and dynamic swimming behaviour of the TlpT mutants were characterised as before. Supplementary Figure S5 shows representative tracks for each strain and a histogram view of the full dataset. Figure 5 shows the free swimming analysis using time spent stopped as the summary measure. As the quadruple mutant was non-motile, the track screening protocol removed most of its tracks and it was excluded. Table 3 summarises the results. 

The single mutants TlpT E296A, TlpT E289A, and TlpT E478A were mostly smooth swimming, spending very little time stopped. TlpT Q485A was stoppier than wild-type, with tracks with shorter runs and more frequent stops. The double mutant TlpT E289A, E296A also had a stoppy phenotype. The three smooth-swimming single mutants, TlpT E296A, TlpT E289A, and TlpT E478A, did not respond to the addition or removal of propionate, constantly rotating throughout the experiment.

The TlpT Q485A mutant stopped when propionate was removed, as seen in wild-type cells, but had a longer adaptation time (134 ± 33 s) than wild-type (40 ± 5 s).

Fewer tethered double mutant TlpT (E289A, E296A) cells rotated than in wild-type. Some stopped cells rotated on addition of propionate, but stopped again before propionate was removed (the inverted adaptive phenotype). The behaviour of the non-motile quadruple mutant (TlpT E289A, E296A, E478A, Q485A) was more extreme than the double mutant. The whole population was non-motile when first tethered. Those cells that responded to propionate addition by beginning rotation all stopped rotating before propionate was removed. There was no response to propionate removal. 

## 3. Discussion

Our results show that adaptation of the cytoplasmic chemoreceptor TlpT is crucial for the chemosensory behaviour of *R. sphaeroides*. Deleting any of the adaptation proteins associated with the membrane or cytoplasmic cluster caused a loss of chemotaxis, showing that both pathways are involved in chemosensory behaviour. Deleting the methylesterase or methyltransferase associated with the membrane-associated pathway resulted in the same phenotype as deleting the equivalent proteins in *E. coli*, suggesting signalling through the membrane cluster is similar to that seen in *E. coli* (1). However, the opposite swimming pattern was seen when deleting the methylesterase or methyltransferase associated with the cytoplasmic cluster. 

Double or quadruple deletions of the *R. sphaeroides* adaption proteins did not return to the wild-type swimming bias, but returned to their stoppy or stopped phenotype. This suggests a much more complex signalling and adaptation process than in *E. coli*. The swimming patterns reflect the signals transmitted through either fully methylated (no CheBs) or fully demethylated (no CheRs) receptors to the CheA kinase. The behaviour of the CheR_3_ deletion mutant, with fully demethylated cytoplasmic receptors, suggests that these receptors either cannot activate the cluster’s kinase (CheA_4_/CheA_3_) or over-activate the cluster’s phosphatase (CheA_3_). The phenotypes of the double and quadruple mutants show that while both pathways are needed for chemotaxis, the cytoplasmic pathway is dominant. 

CheY_6_, which localises to the cytoplasmic cluster and is phosphorylated and dephosphorylated by CheA_3_/A_4_, is the only CheY able to stop the motor. As the extent of phosphorylation is probably governed primarily by the kinase activity of CheA_4_, controlled by TlpT, we investigated the adaptation of this chemoreceptor and the effects on swimming and chemosensory behaviour by mutating putative methylation sites.

We had previously shown that deleting individual MCPs associated with the membrane pathway reduces, but does not halt, the chemotactic response to all chemoeffectors, rather than to specific attractants. TlpT is required for the formation of the cytoplasmic cluster, and deleting TlpC causes a more diffuse cluster structure [17,25]. Simply deleting the receptors thus provides no information about their role in signalling. Our bacterial two-hybrid results showed that the chemoreceptors do interact with the other components of the cluster, and mutagenesis showed that adaptation is critical. 

We tried a range of methods to identify adaptation sites. Initially we used the pattern of amino acids in TlpT to identify glutamates and glutamines potentially involved in adaption, following Alexander and Zhulin’s [20] work with characterised MCPs. Mutations of these candidates caused no change in swimming behaviour (data not shown). As mass spectroscopy has been used to identify modified MCPs [23], we then used MS/MS to identify putative adaptation sites based on patterns of modification in different backgrounds. The method successfully identified the four known adaptation sites on the *E. coli* receptor Tsr with no false positives. It then identified three possible methylation sites in TlpT, E296, E478, and Q485. Bioinformatics informed by these sites identified a fourth putative site, E289.

Wild-type *R. sphaeroides* shows little response to the addition of an attractant, but stops on its removal, followed by adaptation [26,27]. Mutating each glutamate to alanine produced a smooth free swimming phenotype that showed no response to attractant increase or decrease in tethering experiments. Mutating the glutamine site resulted in a more stoppy phenotype that could respond to attractant removal but took much longer to adapt than wild-type cells. Intriguingly, the double mutant TlptT (E289A E296A) was not smooth swimming, but instead more stoppy than wild-type, while the quadruple mutant TlptT (E289A E296A E478A Q485A) was essentially non-motile. The stopped cells of the double mutant responded to propionate addition in tethering experiments and similarly the tethered non-motile quadruple mutant cells started to spin on addition of propionate, rapidly returning to stopped before removal of propionate. This pattern of swimming on propionate addition, followed by a return to the stopped state, is consistent with adaptation to propionate addition. The membrane chemosensory signalling and adaptation pathway is still active in these mutants, suggesting that the response and adaptation seen is through the membrane-localised pathway.

The range of phenotypes for different combinations of methylation site mutants indicates that TlpT is a true chemoreceptor and is tuned by the cytoplasmic-pathway-associated methyltransferase and methylesterase (CheR_3_ and CheB_2_). However, this adaptation process appears very different from that of better-characterised *E. coli* MCPs, where mutation of single glutamates does not produce a strong phenotype. The pattern and diversity is more similar to that seen in *B. subtilis* McpB [28]. While the results clearly show that E296, E289, and E478 are adaptation sites, it is less clear whether Q485 is an adaptation site. Mutating the three glutamates individually resulted in non-chemotactic smooth swimming cells, but mutation of Q485 resulted in a subtle phenotype. As we were unable to produce triple glutamate or double glutamate-glutamine mutants, we cannot say whether the extreme phenotype of the quadruple mutant is the result of the three glutamate-to-alanine mutations or also requires the glutamine mutation. 

When interpreting the mutant phenotypes, it is important to remember that the membrane-associated chemosensory cluster and its associate methyltransferase and methylesterase (CheR_2_ and CheB_1_) were present and able to signal in response to change. The results of the double and quadruple TlpT mutant and CheB_2_ deletion mutant suggest that the chemosensory cluster produced an increased concentration of CheY_6_~P. 

Receptors that had two of the three glutamate adaption sites available for methylation had a phenotype similar to the CheR_3_ deletion mutants. Removing all three sites changed the swimming behaviour towards a stopped phenotype, which suggests an increase in the amount of steady-state CheY_6_~P. Addition of an attractant caused a transient displacement of CheY_6_~P from the motor. It seems probable that the overall steady-state level remained high, with the cytoplasmic cluster continuing to generate high levels CheY_6_~P and the transient displacement of CheY_6_~P and associated brief motor rotation the result of a pulse of CheY_3_~P or CheY_4_~P from the membrane-associated cluster, followed by rapid adaptation of the membrane receptors. 

This picture fits with the behaviour of the CheB_1_ and CheR_2_ mutants. The smooth swimming of the CheB_1_ deletion mutant suggests that fully methylated membrane receptors produce high levels of CheY_3_/Y_4_~P and displace CheY_6_~P from the motor. In contrast, the demethylated membrane receptors of the CheR_2_ deletion mutant were unable to signal, leaving the motor occupied by CheY_6_~P from the cytoplasmic receptors and resulting in stoppy swimming.

Our understanding of *R. sphaeroides* chemotaxis is as yet incomplete, but it is clear that signals through the two pathways interact to produce a balanced response. The cytoplasmic cluster produces the strongest steady-state signal, with the major chemoreceptor, TlpT, adapting to the cytoplasmic state and controlling CheA_4_ kinase activity to produce the steady-state level of CheY_6_~P. The membrane-associated cluster is structurally similar to that of *E. coli* and senses changes in nutrient concentration in the external environment. It may produce CheY_3_/Y_4_~P on attractant addition to compete CheY_6_~P off the motor and allow a period of smooth swimming. 

It seems likely that TlpT senses the cell’s internal energy state and that long-term adaptation of the cytoplasmic receptors tunes the steady-state response. This hypothesis is supported by the ease with which methylated TlpT peptides were isolated for MS/MS: the cells were in a stimulated environment for significantly longer than in similar in vitro techniques and methylated Tsr residues were less common than methylated TlpT residues, suggesting greater stability. This hypothesis may also explain the split CheA histidine kinase. CheA_3_ has the histidine domain, but lacks the kinase domain. The kinase domain on CheA_4_ is linked to a P5 domain that only interacts with TlpT, suggesting the activity of the kinase domain is controlled by signals coming through this adaptable cytoplasmic chemoreceptor. The phosphatase domain of CheA_3_, which dephosphorylates CheY_6_~P, has a P5 domain that interacts with TlpC, which has no obvious adaptation sites. Kinase activation is thus separated from its steady dephosphorylation, potentially tuning the steady-state level of the motor-stopping CheY_6_~P.

We think, taking our current data with previous data, that TlpT responds and adapts to the current metabolic state and through CheA_4_ kinase activity keeps the CheY_6_~P level stable. CheY_3_~P and/or CheY_4_~P produced by the membrane cluster allow periods of smooth swimming by competing with CheY_6_~P for the motor switch protein FliM. On attractant reduction, the level of CheY_3_/Y_4_~P is reduced, CheY_6_~P binds the motor and the motor stops, before adaptation of the membrane receptors allows some CheY_3_/Y_4_~P and the motor returns to swim-stop motility. A combination of long-term signalling and adaptation through the cytoplasmic cluster and transient responses through the membrane cluster allows *R. sphaeroides* to tune responses relative to its metabolic need.

## 4. Materials and Methods

### 4.1. Strains

Strains used in this study are listed in Table 4, below.

### 4.2. Growth Conditions

*R. sphaeroides* was grown aerobically in the dark in succinate medium at 30 °C with 225 rpm shaking, until mid-exponential growth was reached (OD_700nm_ ~ 0.5). 

### 4.3. Molecular Genetics Techniques

Standard genetic techniques were used. Pfu polymerase was used for all PCRs. Primers were synthesised by Sigma-Genosys. DNA was extracted with plasmid mini-prep kits (QIAGEN, Manchester, UK) and sequenced by Source Bioscience. DNA sequences were analysed using Clone Manager 9.

### 4.4. Bacterial Two-Hybrid Screening

*R. sphaeroides* cytoplasmic cluster chemotaxis genes were each cloned into four bacterial two-hybrid plasmids to obtain N- and C-terminal fusions of the T18 and T25 tags to each protein (Karimova 2001, Karimova 2005). The resulting plasmid library is listed in Table 5.

Signal detection used LB agar plates containing ampicillin and kanamycin to select cells containing both the T18 and T25 constructs. IPTG was added to induce expression and X-Gal to detect beta-galactosidase activity. Plates were incubated at 30 °C for 48 h, then room temperature for a week. As the bacterial adenylate cyclase-based two-hybrid (BACTH) system has heterogeneity due to cAMP expression regulation, the assay cultures were inoculated with several co-transformation colonies. Each interaction was tested in at least two independent assays performed on different days, from different co-transformations.

A control assay was run for every construct in the library using cotransformants of the plasmid in question with an empty plasmid expressing just complementary tag. All plasmids produced negative results when tested against complementary tags alone. Positive and negative controls were also carried out for every interaction assay. The negative control was a co-transformant of two empty bacterial two-hybrid plasmids with complementary tags. The positive controls were the pT18-zip and pKT25-zip plasmids coding for a strongly interacting leucine zipper motif [35].

### 4.5. Chemoreceptor Purification

C-terminal His-tagged TlpT was expressed in *R. sphaeroides* WS8N, JPA2365, and JPA2366 from plasmid pIND4-tlpT. C-terminal His-tagged Tsr was expressed in *E. coli* RP437 (wild-type) and RP1091 (RP437 gutted of all chemotaxis genes) from plasmid pQE60-tsr. Stationary culture was diluted 1 in 50 in 6 l medium (*R. sphaeroides*: succinate medium; *E. coli*: 2TY medium), immediately induced with 2.5 µM IPTG and grown with 225 rpm shaking to mid-log phase (*R. sphaeroides*: OD_700nm_ = 0.6, *E. coli*: OD_600nm_ = 0.5). Culture was then incubated with 100 µM L-methionine and 30 µg/mL chloramphenicol for 30 min. 100 µM attractant was added (*R. sphaeroides*: propionate, *E. coli*: L-serine), the culture incubated for 20 min, and the cells harvested by centrifugation at 6000 g, 4 °C, 30–40 min. Cell pellets were immediately frozen and stored at −80 °C. 

Cell pellets were thawed on ice and chemoreceptor purified by nickel affinity chromatography under denaturing conditions following the manufacturer’s protocol (Bio-Rad, city, province abbreviation, country). Thawed cell pellets were resuspended in denaturing protein buffer (5 mL/g cell pellet, 10 mM imidazole, 100 mM NaH_2_PO_4_, 10 mM Tris-HCl, 8 M urea, pH 8.0; 0.2% Tween for Tsr, 0.02% Tween for TlpT) with gentle stirring at room temperature. Cells were lysed by sonication on ice (4 min sonication time, pulsed 5 s on, 15 s off, Vibracell sonicator). Cell lysate was centrifuged at 10 000 g for 30 min at room temperature. Supernatant was incubated with 1 mL Ni-NTA slurry on an orbital shaker for 1 h at room temperature. The resin was collected by centrifugation at 1000× *g* for 10 min at room temperature. The supernatant was discarded, wash buffer (denaturing protein buffer, pH 6.3 with HCl) added, and incubated for 1 h as before. A second wash step was performed as described, then buffer removed. The resin was resuspended in 5 mL wash buffer and the slurry poured into a sealed chromatography column and allowed to settle. The remaining wash buffer was run through the column, the protein eluted with 5 mL denaturing protein buffer at pH 5.9, followed by 5 mL buffer at pH 4.5, collecting 1 mL fractions. Protein usually eluted within the first two fractions of both elution buffer. Fractions with protein were identified with the Bradford assay, pooled, and, in the case of TlpT, concentrated with a centrifuge concentrator. All of the protein solution was then subjected to gel electrophoresis and bands corresponding to the chemoreceptor were excised, pooled, and sent for MS/MS on ice. This protocol was repeated twice (*E. coli*) / three times (*R. sphaeroides*) for each background strain.

### 4.6. MS-MS

Following SDS-PAGE, TlpT gel bands were reduced with 10 mM DTT and alkylated with 50 mM iodoacetmaide as described before [36]. Proteins were then digested with elastase in 50 mM ammonium bicarbonate at 37 °C overnight. After extraction from the gel and C18 desalting, peptides were analysed on an nLC-MS/MS system consisting of a nAcquity nano-HPLC (Waters Corp., Milford, MA, USA ) and an Orbitrap Velos mass spectrometer (Thermo Fisher Scientific Inc., Waltham, MA, USA). Chromatographic separation was achieved on a BEH C18 column (75 µm × 250 mm, 1.7 µm particles, Waters) using a gradient of 3–40% acetonitrile in 0.1% formic acid within 60 min at 250 nL/min. Precursor masses were acquired in the Orbitrap detector at 60,000 resolution and an AGC target of 1E6. MS/MS spectra of selected precursors were acquired in the linear ion trap after collision-induced-dissociation at a normalised collision energy of 35%. Spectra were searched against a database of *R. sphaeroides* proteins (4193 entries, retrieved in 2012) using Mascot Version 2.3 (Matrix sScience Inc., Boston, MA, USA) with a mass tolerance of 5 ppm for precursors and 0.5 Da for MS/MS fragments. Glutamine deamidation and glutamate and glutamine methylation were set as variable modifications and carbamidomethylation of cysteine as fixed modification. The false discovery rate was adjusted at 1% at peptide level. Peptides and proteins were quantified using Progenesis QI for Proteomics (Waters) to determine their relative abundances across samples. 

### 4.7. Site-Directed Mutagenesis

Overlap extension PCR was used to generate constructs to mutate TlpT. The constructs consisted of a fragment of the TlpT gene with the desired mutation with 500 bp up and downstream of the mutation. The constructs were inserted into the allelic exchange suicide vector pK18*mobsacB* and the resulting mutation plasmids transformed into *E. coli*, then conjugated into the desired *R. sphaeroides* strain. Allelic exchange via recombination was selected for over two selection rounds using naladixic acid and sucrose as previously described [37]. Four point mutations were made, singly and in combination.

Similarly, a deletion plasmid was constructed to delete *cheBRA* from strains with deletions of methylesterase and methyltransferase homologues and an insertion plasmid was constructed to insert *yfp* onto the N-terminus of *cheW_4_* in each of the TlpT mutation strains.

### 4.8. Free Swimming Analysis

Motile *R. sphaeroides* cells were harvested by centrifugation of 100 µl aliquots at 1000 rpm for 1 min. The cell pellet was gently resuspended in 1 mL PIPES motility buffer (10 mM PIPES, 30 µg/mL chloramphenicol, pH 7.2). The sample was left to equilibrate for 15 min. A 0.2 × 2 mm capillary tube was filled with equilibrated cells, sealed with silicon grease to create a zero-flow environment and placed lengthwise on a glass slide. The centre of the capillary was visualised under 20 × phase contrast microscopy (Nikon Optiphot phase contrast Microscope, Japan). The field of view was held constant and cells swum freely in and out of view. The image was recorded for 2 min at 20 ms per frame, 6 µs exposure per frame (digital DALSA Genie-HM640 camera, New York, USA). Three recordings of fresh capillaries were made. The experiment was repeated with three biological replicates per strain, giving 18 min of recordings. 

**Figure 6:**
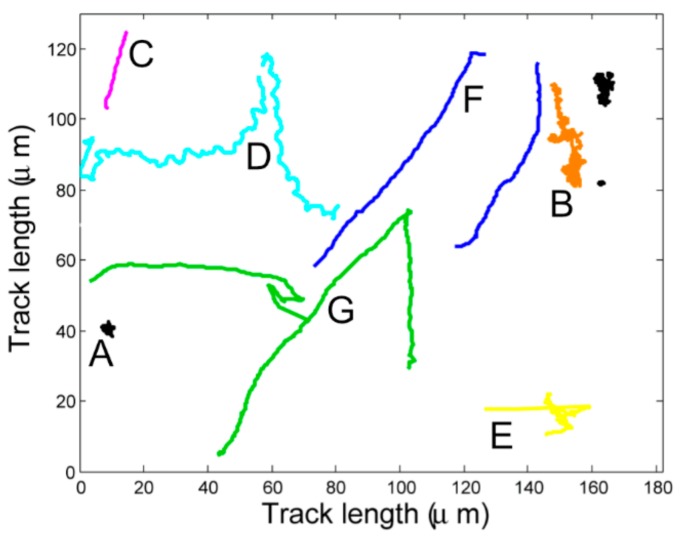
Representative free swimming tracks. (**A**) non-motile (black), (**B**) drifting: non-motile or dead cells that appear to move slightly, due to Brownian motion or buffeting by mobile cells (orange), (**C**) very short or truncated tracks due to cells moving at an angle to the plane of the field (magenta), (**D**) corkscrews: a cell rotating in such a way as to rock its centre from side to side (cyan), (**E**) jumps: software incorrectly assigning two tracks to be from the same object, leading to an inconceivably large jump between frames (yellow), (**F**) smooth swimming tracks (blue), and (**G**) wild-type run-stop-run (green).

The movies were analysed using the software Tracker [38]. Briefly, each frame of a movie was considered separately. Pixel intensity was used to identify all of the objects on the frame. The relationships between each successive frame’s objects were determined using Gaussian probability density functions to form tracks. Tracks were recorded as a series of xy coordinates through time, removing dependency on the video and initial frame. Representative tracks obtained are shown in Figure 6.

The data were censored to remove problematic tracks. Dead and other non-motile cells were removed using minimum bounded radius (MBR) 9 µm. Tracks erroneously formed of different objects (jumps) were removed using a maximum frame to frame speed of 90 µm/s, which is twice the *R. sphaeroides* top speed. Tracks formed of cells moving at an extreme angle to the field of view (truncated) were removed using a minimum track length of 50 frames, or 1 s. Of the remaining tracks, the 10% most tortuous tracks, using the median absolute curvature to define tortuosity, were removed, thus removing corkscrews. 

The censored tracks were classified into runs and stops using the RunStopAnalysis software [39]. Stopped strain JPA1216 was used to produce a reference set of stops and smooth-swimming strain JPA1353 was used to produce a reference set of runs. Each frame-to-frame transition of each track in a mutant strain was compared to the two reference sets and the probability of its identity as a run or a stop determined. The minimum length of a run and a stop was set at two frames, with stops taking precedence over runs. 

The classified tracks were summarised by the fraction of time spent stopped. The sets of summary statistics for all of the strains examined were compared by a Krusal-Wallis test with post-hoc Dunn pair-wise comparison test.

### 4.9. Tethered Cell Analysis

Motile *R. sphaeroides* cells were harvested by centrifugation of 1 mL aliquots at 1000 rpm for 1 min. The cell pellet was washed twice with 1 mL PIPES motility buffer (10 mM PIPES, 30 µg/mL chloramphenicol, pH 7.2), centrifuging after each wash, then resuspended in 1 mL motility buffer. 10 μL resuspended culture was incubated with 2 µL anti-flagellum antibody on a 12 mm diameter circular glass slide for 20 min, then sealed into a flow cell, as described in [40]. The flow cell was visualised using phase contrast microscopy at 40 × magnification and a field of view with ideally 3–10 motile tethered cells chosen. The flow cell was subjected to 3 min motility buffer, 5 min motility buffer with 100 µM propionate (attractant), and 5 min motility buffer. The flow cell was visualised and recorded using the same set-up as for free swimming, collecting 13 min of 100 Hz video per repeat. One field of view was recorded for each flow cell. Three flow cells were recorded for each strain at a time. This was repeated three times, giving nine repeats for each strain.

The videos were analysed using BRAS and Click&Mean, as described in [40]. All cells that rotated at some point in the video and that remained in position were analysed. Cells were classified by eye as always rotating despite changes in environment (unresponsive), responding to the addition of attractant by beginning rotation, usually losing rotation before attractant is removed (inverted adaptive), responding to the removal of attractant by stopping but not adapting to restart (responsive), or responding to stimulus by stopping, then restarting again (adaptive). Adaptation was defined as a period of clear rotation for at least 5 s after a prolonged stop. Cells were also classified as either having a ‘stoppy’ appearance or a constant rotation signal. For cells showing adaptation, the length of the stop was summarised as mean and standard deviation. 

## Figures and Tables

**Table 1:** Bacterial two-hybrid assay results showing interactions between cytoplasmic cluster proteins tagged on N and C terminals after 24 h incubation (+), between 24 and 72 h incubation (++), and after 72 h incubation (+++). Empty squares indicate no interaction within 72 h.

			T25									
			CheA_3_		CheA_4_		CheW_4_		TlpC		TlpT	
			N	C	N	C	N	C	N	C	N	C
T18	CheA_3_	C	++	+	++		+		++			
		N										
	CheA_4_	C	++		+++	+++	+++					
		N			+++		+++					
	CheW_4_	C	++	+	++		+	+++	+++	+++		
		N	++	+	++		+		+++	+		++
	TlpC	C					+++		++			
		N	+++				+++	+	+	++		
	TlpT	C	+									
		N	+		+		++					++

**Table 2:** Free swimming and tethering phenotypes for CheB and CheR deletion mutants. In all populations tested, a small percentage of cells showed no response under any condition.

Strain	Free Swimming (Steady State)		Tethered (Response to Attractant Addition and Removal)	
	Tracks analysed after censoring	Phenotype	Cells analysed	Predominant tethering phenotype
Wild-type	1532	Reference standard	63	Respond and adapt to removal
*ΔcheB_1_*	1057	Smooth	80	Unresponsive
*ΔcheB_2_*	1878	Stoppy	25	Inverted adaptive
*ΔcheR_2_*	1450	Stoppy	82	Unresponsive
*ΔcheR_3_*	609	Smooth	59	Unresponsive
*ΔcheB_1_, cheR_2_*	613	Stoppy	29	Unresponsive
*ΔcheB_2,_cheR_3_*	730	Smooth	17	Unresponsive
*ΔcheB_1,2,_cheR_1,2,3_*	559	Smooth	24	Unresponsive

**Table 3:** Free swimming and tethering phenotypes for TlpT methylation site mutants.

Strain	Free Swimming (Steady State)		Tethered (Response to Attractant Addition and Removal)	
	Tracks analysed after censoring	Phenotype	Cells analysed	Predominant tethering phenotype
*tlpT* E296A	884	Smooth	25	Non-responsive
*tlpT* E289A	524	Smooth	14	Non-responsive
*tlpT* E289A E296A	484	Stoppy	14	Inverted adaptive (stopped cells responded by rotating and adapted to propionate addition)
*tlpT* E478A	468	Smooth	24	Non-responsive
*tlpT* Q485A	505	Stoppy	11	Adaptive (responded to propionate removal, slower to adapt than wild-type)
*tlpT* E289A E296A Q485A E478A	3988 non-motile tracks	Stopped	10	Inverted adaptive (stopped cells responded by rotating and adapted to propionate addition)

**Table 4:** Bacterial strains used in this study.

Strain	Genotype	Origin
*Escherichia coli*		
RP437	Wild-type	[29]
RP1091	RP437 gutted of all chemotaxis genes	[22]
DHM1	Strain for bacterial two-hybrid assay	[30]
*Rhodobacter sphaeroides*		
WS8N	Naladixic acid resistant derivative of wild-type WS8	[31]
JPA517	WS8N Δ(*cheB_1_*)	[14]
JPA565	WS8N Δ(*cheR_2_*)	[14]
JPA1216	WS8N *cheY_6_*(D57A)	[13]
JPA1320	WS8N Δ(*cheR_3_*)	[12]
JPA1323	WS8N Δ(*cheB_2_*)	[12]
JPA1331	WS8N Δ(*tlpT*)	[12]
JPA1353	WS8N gutted of all chemotaxis genes	[13]
JPA1377	WS8N Δ(*cheB_2_, cheR_3_*)	This study
JPA1378	WS8N ∆(*cheB_1_, cheR_2_*)	This study
JPA1379	WS8N ∆(*cheB_1,2_, cheR_1,2,3_*)	This study
JPA2365	WS8N Δ(*cheB_1_, cheB_2_, cheBRA*)	This study
JPA2366	WS8N Δ(*cheR_1_, cheR_2_, cheR_3_, cheBRA*)	This study
JPA2332	WS8N *tlpT*(E296A)	This study
JPA2367	WS8N *tlpT*(E289A)	This study
JPA2368	WS8N *tlpT*(E289A, E296A)	This study
JPA2369	WS8N *tlpT*(E478A)	This study
JPA2370	WS8N *tlpT*(Q485A)	This study
JPA2371	WS8N *tlpT*(E289A, E296A, E478A, Q485A)	This study
JPA2378	WS8N *yfp-cheW_4_ tlpT*(E289A, E296A)	This study
JPA2379	WS8N *yfp-cheW_4_ tlpT*(E478A)	This study
JPA2380	WS8N *yfp-cheW_4_ tlpT*(Q485A)	This study
JPA2381	WS8N *yfp-cheW_4_ tlpT*(E289A, E296A, E478A, Q485A)	This study

**Table 5:** Plasmids used in this study.

Plasmid	Description	Source
pIND4	Protein expression vector for *E. coli* and *R. sphaeroides*, kanamycin resistant, inducible *lac* promoter upstream of various restriction enzyme sites	[32]
pQE60	Protein expression vector, Q-terminus 6xHis tag, inducible *lac* promoter	QIAGEN
pK18*mobsacB*	Allelic exchange suicide vector	[33]
pKT25	IPTG-inducible *E. coli* expression vector for BACTH assay, kanamycin resistance, N-terminal T25 tag	[34]
pKT25 derivatives	pKT25 containing *cheA_3_*, *cheA_4_*, *cheW_4_*, *tlpC*, or *tlpT*	This study
pKNT25	IPTG-inducible *E. coli* expression vector for BACTH assay, kanamycin resistance C-terminal T25 tag	[30]
pKNT25 derivatives	pKNT25 containing *cheA_3_*, *cheA_4_*, *cheW_4_*, *tlpC*, or *tlpT*	This study
pUT18	IPTG-inducible *E. coli* expression vector for BACTH assay, ampicillin resistance C-terminal T18 tag	[34]
pUT18 derivatives	pUT18 containing *cheA_3_*, *cheA_4_*, *cheW_4_*, *tlpC*, or *tlpT*	This study
pUT18C	IPTG-inducible *E. coli* expression vector for BACTH assay, ampicillin resistance N-terminal T18 tag	[34]
pUT18C derivatives	pUT18C containing *cheA_3_*, *cheA_4_*, *cheW_4_*, *tlpC*, or *tlpT*	This study

## Supplementary Materials

Supplementary materials can be found at https://www.mdpi.com/1422-0067/20/20/5095/s1.
