# Supplementary material for: Mechanism of Signalling and Adaptation through the Rhodobacter sphaeroides Cytoplasmic Chemoreceptor Cluster
Source: Int J Mol Sci. 2019 Oct 14;20(20):5095. doi: 10.3390/ijms20205095 (PMC6829392; doi:10.3390/ijms20205095)
Supplement: Supplementary file 1 [file ijms-20-05095-s001.pdf]

Supplementary information

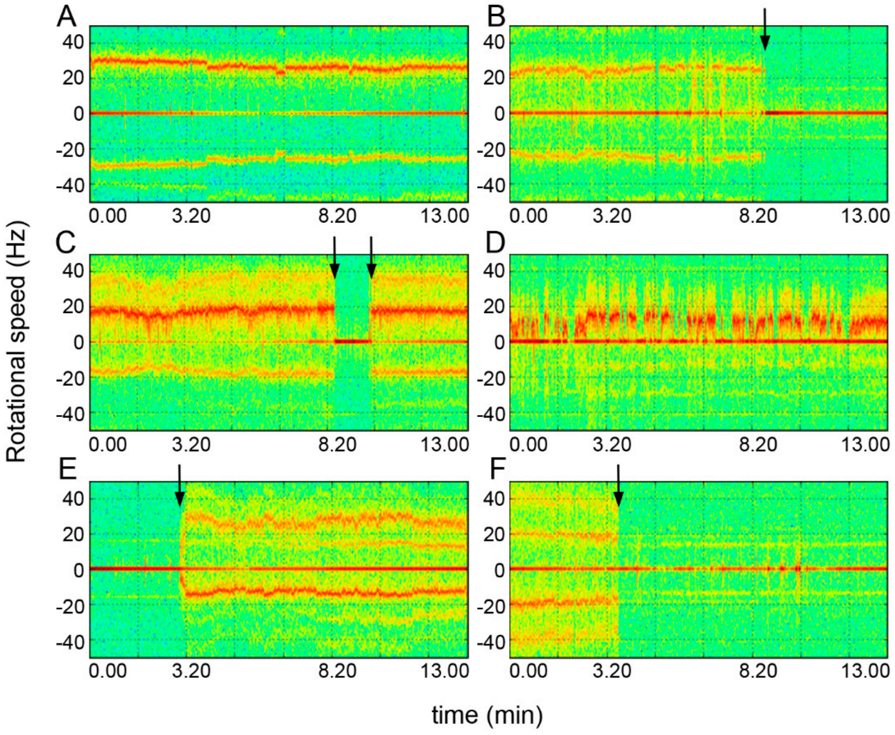

Supplementary Figure S1: Representative BRAS Click&Mean outputs from tethered cells showing events of interest. Attractant (proprionate) was added at 3min and removed at 8min. A. Unresponsive: rotation does not change with attractant addition or removal. B. Responsive: rotation stops on attractant removal. C. Adaptive: rotation stops and starts again in response to change in attractant. D. Stoppy rotation regardless of attractant state. E. Inverted adaption: rotation begins on attractant addition. F. Stop on addition of attractant (rare phenotype).

Supplementary Table S1: Numbers of modified and unmodified peptides identified at any abundance in fragmented Tsr, collected from wild-type *E. coli* or *E. coli* gutted for chemotaxis genes, for any glutamate or glutamine that was found to be modified at any abundance

**Methylation on glutamate sites**				
**Site**	**Total peptides**	**Methylated peptides**	**Percentage modified**	
**E304**	**20**	**2**	**10**	
E321	*11*	*2*	*18.18*	
E325	*8*	*2*	*25*	
E458	14	1	7.14	
E479	55	1	1.82	
**E492**	**40**	**15**	**37.5**	
**E493**	**40**	**5**	**12.5**	
**Deamidation and methylation on glutamine sites**				
**Site**	**Total peptides**	**Deamidated peptides**	**Deamidated and methylated peptides**	**Percentage modified**
Q48	18	1	0	5.56
Q78	46	1	0	2.17
Q154	24	1	0	4.17
Q173	26	1	0	3.85
**Q297**	**37**	**8**	**1**	**24.32**
**Q311**	**28**	**17**	**1**	**64.29**
Q318	*12*	*2*	*2*	*33.33*
Q338	19	0	1	5.26
Q408	21	1	0	4.76
Q466	16	0	1	6.25
Q485	54	2	1	5.56
Q486	53	1	2	5.66
Q504	14	1	0	7.14
Q520	23	1	0	4.35

Bold residues are known methylation sites in *E. coli* Tsr and their glutamate pair partners

Italicised residues are non-adaptation sites found to be modified above 10%

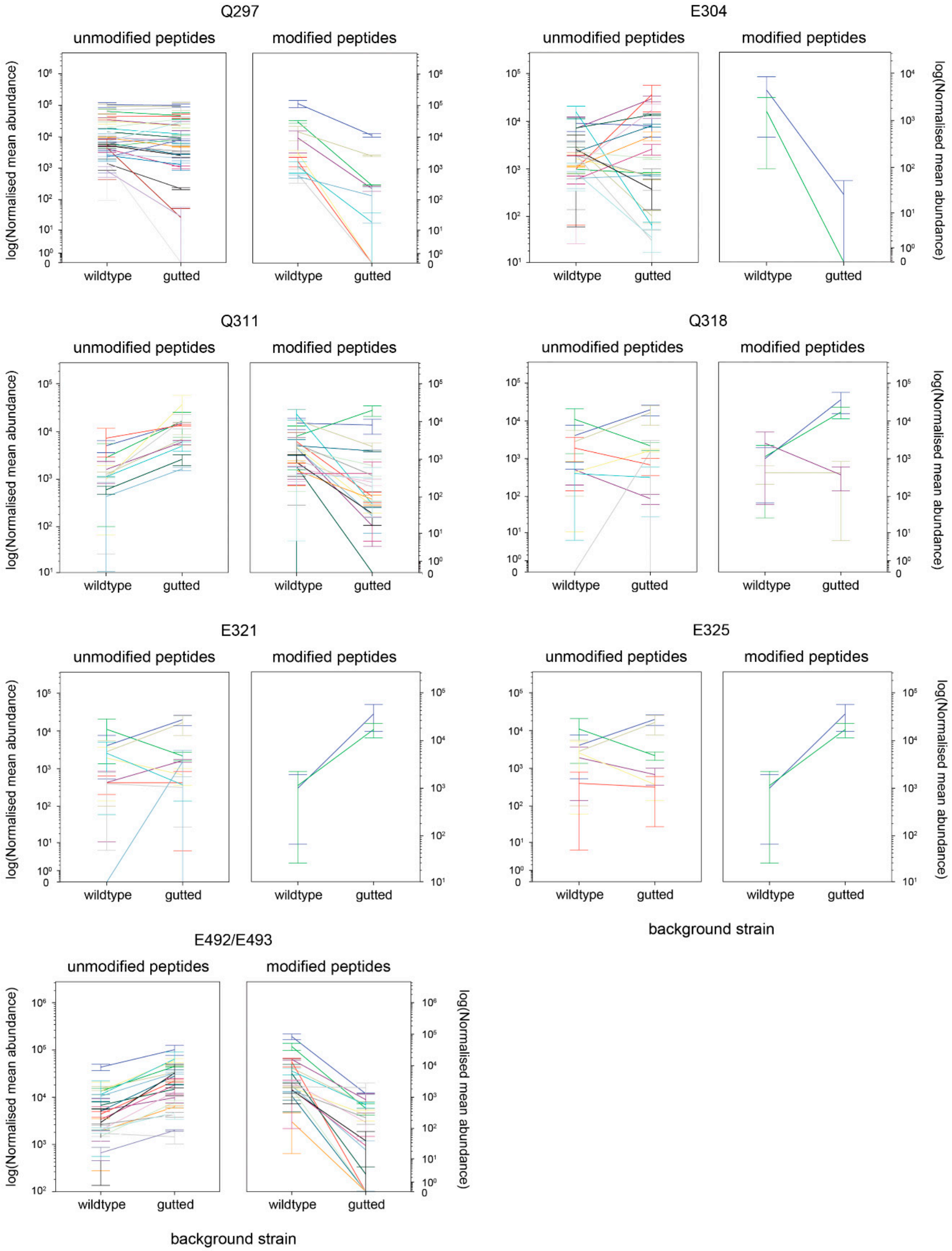

Supplementary Figure S2: Relative abundance of each *E. coli* Tsr peptide (modified or unmodified) in the two backgrounds, RP437 (wildtype) and RP1091 (gutted of chemotaxis genes), for glutamate and glutamine sites with modifications in over 10% of identified peptides from MS/MS. Sites E492 and E493 are combined, as it is

difficult to resolve data for a glutamate pair. Each measurement was done in duplicate, with error bars showing the two values. Each colour within a plot shows a particular peptide containing that residue.

Supplementary Table S2: Number of modified and unmodified peptides identified at any abundance in fragmented TlpT, collected from a wildtype, no CheB, or no CheR background, for any glutamate or glutamine that was found to be modified at any abundance

Methylation on glutamate sites				
Site	Total peptides	Methylated peptides	Percentage modified	
E30	28	3	10.71	
E32	28	1	3.57	
E96	40	2	5.00	
E146	13	3	23.08	
E170	44	34	77.27	
E296	56	16	28.57	
E435	38	1	2.63	
E450	37	1	2.70	
E478	73	23	31.51	
E521	104	1	0.96	
Deamidation and methylation of glutamine sites				

Site	Total peptides	Deamidated peptides	Deamidated and methylated peptides	Total percentage modified
Q149	34	0	3	8.82
Q183	42	2	1	7.14
Q442	45	1	0	2.22
Q451	37	0	1	2.70
Q457	17	0	2	11.76
Q485	103	42	0	40.78
Q487	91	0	1	1.10
Q525	78	1	0	1.28
Bold residues were modified in more than 5% of peptides				

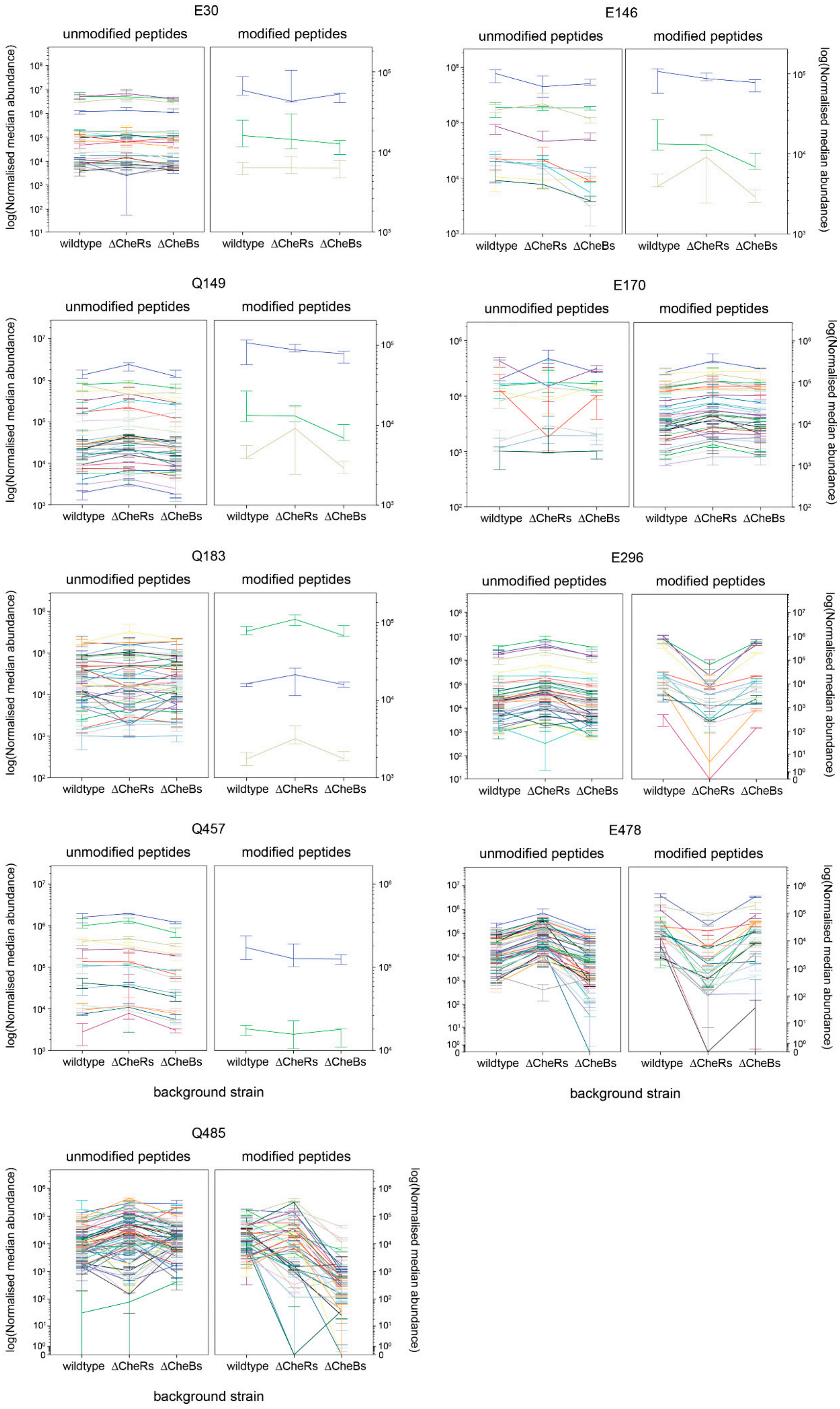
E30
unmodified peptides
modified peptides
E146
Q149
E170
Q183
E296
Q457
E478
Q485
log(Normalised median abundance)
wildtype
ΔCheRs
ΔCheBs
background strain

Supplementary Figure S3: Relative abundance of each *R. sphaeroides* TlpT peptide (modified or unmodified) in the three backgrounds, WS8N (wildtype), JPA3265 (all CheB homologues deleted) and JPA2366 (all CheR homologues deleted), for glutamate and glutamine sites with modifications in over 5% of identified peptides. Each measurement was done in triplicate, with the median shown and error bars showing min and max. Each colour within a panel represents one peptide.

Supplementary Table S3: Cytoplasmic cluster foci observed in TlpT mutant strains. The labelled cytoplasmic cluster component YFP-CheW4 was observed using phase-contrast fluorescence microscopy.

Strain no	Genotype	Cells analysed	Cells with foci		Cells without foci	
			No.	%	No.	%
JPA2378	tlpT E478A yfp-cheW4	190	182	95.7	8	4.3
JPA2379	tlpT E289A E296A yfp-cheW4	184	178	96.7	6	3.3
JPA2380	tlpT Q485A yfp-cheW4	159	151	94.9	8	5.1
JPA2381	tlpT E289A E296A Q485A E478A yfp-cheW4	191	180	94.2	11	5.8

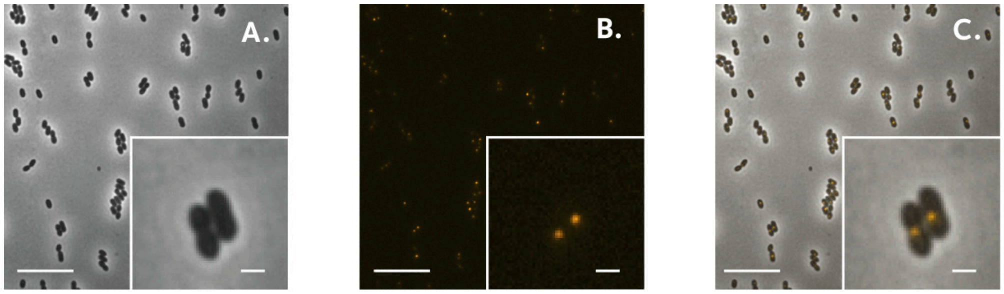

Supplementary Figure S4. Representative images for cytoplasmic cluster foci in TlpT mutant strains. A) Phase-contrast image of the cells; B) Fluorescence image of the same field of view (YFP channel); C) Overlay of the first two images. The scale bars represent 10 μm in the big image and 1 μm in the inset.

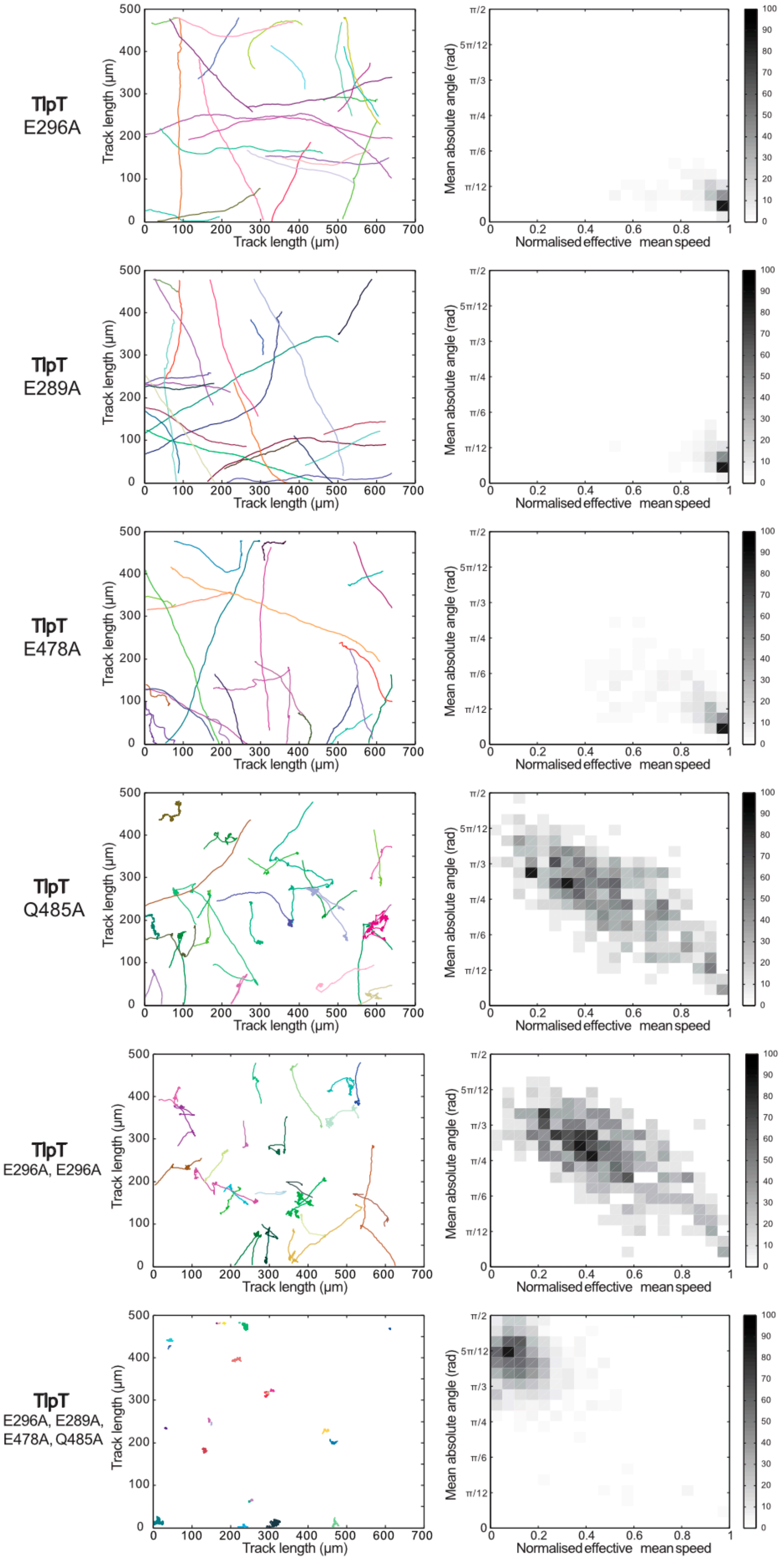
TlpT
E296A
TlpT
E289A
TlpT
E478A
TlpT
Q485A
TlpT
E296A, E296A
TlpT
E296A, E289A,
E478A, Q485A
Track length (µm)
Track length (µm)
Mean absolute angle (rad)
Normalised effective mean speed
π/2
5π/12
π/3
π/4
π/6
π/12
0
0.2
0.4
0.6
0.8
1
0
100
200
300
400
500
600
700

Supplementary Figure S5: Free swimming analysis of TlpT mutants. A view of 25 tracks after censoring (left panel). 400 bin two-dimensional histograms summarising the full set of tracks for each strain using mean absolute angle (MAC) vs normalised effective mean speed (NEMS). Smoother tracks have low MAC and high NEMS, while tracks with more time spent stopped have high MAC and low NEMS.
